# Fast Air-to-Liquid Sampler Detects Surges in SARS-CoV-2 Aerosol Levels in Hospital Rooms

**DOI:** 10.3390/ijerph20010576

**Published:** 2022-12-29

**Authors:** Cristina del Álamo, Ángela Vázquez-Calvo, África Sanchiz, Gil Rodríguez-Caravaca, Rocío Martín, Bruno Hernáez, Pablo Méndez-Vigo-Carranza, Juan Sánchez García-Casarrubios, Antonio Alcamí, José Luis Pérez-Díaz

**Affiliations:** 1 Escuela Politécnica, Universidad de Alcalá, 28801 Alcalá de Henares, Spain; 2 Centro de Biología Molecular Severo Ochoa (CBMSO), Consejo Superior de Investigaciones Científicas (CSIC), and Universidad Autónoma de Madrid (UAM), 28049 Madrid, Spain; 3 Preventive Medicine Service, Hospital Universitario Fundación Alcorcón, 28922 Alcorcón, Madrid, Spain; 4 Department of Medical Specialities and Public Health, Universidad Rey Juan Carlos, 28922 Alcorcón, Madrid, Spain; 5 COUNTERFOG S.L., 28341 Valdemoro, Madrid, Spain

**Keywords:** airborne, fast sampling, viruses, SARS-CoV-2, liquid sample, disease control, environmental health

## Abstract

The COVID-19 pandemic highlighted the dangers of airborne pathogen transmission. SARS-CoV-2 is known to be transmitted through aerosols; however, little is known about the dynamics of these aerosols in real environments, the conditions, and the minimum viral load required for infection. Efficiently measuring and capturing pathogens present in the air would help to understand the infection process. Air samplers usually take several hours to obtain an air sample. In this work a fast (1–2 min) method for capturing bioaerosols into a liquid medium has been tested in hospital rooms with COVID-19 patients. This fast sampling allows detecting transient levels of aerosols in the air. SARS-CoV-2 RNA is detected in aerosols from several hospital rooms at different levels. Interestingly, there are sudden boosts of the SARS-CoV-2 load in the air, suggesting that SARS-CoV-2 could be released abundantly at certain moments. These results show that the distribution of SARS-CoV-2-containing aerosols is not homogeneous in the hospital room. This technology is a fast and effective tool for capturing airborne matter in a very short time, which allows for fast decision-making any kind of hazard in the air is detected. It is also useful for a better understanding of aerosols dynamics.

## 1. Introduction

As in many respiratory diseases, airborne transmission is the main transmission channel of COVID−19 [1], although droplet, contact, and fecal-oral transmission have also been reported [2,3]. Epidemiological studies of SARS-CoV-2 are also consistent with aerosol transmission [4,5]. The main modes of transmission involve the exhalation of viral particles from the respiratory tract via large particles or droplets, which fall by gravitational forces; or smaller particles or aerosols, which remain airborne for prolonged periods of time with the potential to travel greater distances [6]. Particles are classified as droplets when they have a diameter greater than 5 μm and as aerosols when it is less than 5 μm [7]. However, particles up to 20 μm with favorable aerodynamic characteristics and low settling velocity or by quickly evaporating may remain longer in the air, behaving as aerosols in suspension [6,8,9]. Based on this aerodynamic behavior, some studies have even suggested establishing the size distinction between aerosol and droplets as 100 μm [1,10].

Understanding the behavior of virus-laden droplets and aerosols is critical for mitigating of the spread of airborne infectious diseases [6]. However, SARS-CoV-2 transmission dynamics remains essentially unknown.

Our knowledge of many parameters associated with the SARS-CoV-2 is still very limited, including critical infectious viral load in droplets and aerosols, and dose-response characteristics of the virus in humans. As a reference, the estimated infectivity (ID_50_) for SARS-CoV (the original SARS virus) is estimated, through a dose-response model in transgenic mice, at 280 plaque forming units by intranasal infection [11]. Each SARS-CoV-2 virion is approximately 60–140 nm in diameter [12,13]. Aerosolized particles smaller than 5 µm can potentially harbor quite a few viral particles and remain airborne for several hours [6].

Over the years, several methodologies for sampling airborne agents have been developed [14]. The most common are liquid impactors (e.g., swirling collector), and solid impactors (e.g., cyclone samplers) as well as filters [15]. These devices are less efficient for collecting the finest airborne particles. Only a few of them have succeeded detecting the presence of SARS-CoV-2 RNA in aerosols [16,17,18]. The finest aerosols are precisely the most dangerous as they remain airborne much longer and are more difficult to filter.

The development of faster and more efficient air sampling methods can help to better understand the dynamics of virus-containing aerosols and could be a useful tool in fighting pandemics. Any tool providing fast information on the concentration of airborne virions, or just detecting their presence, will provide essential information to avoid shooting in the dark against virus transmission.

In this way, a novel fast air-to-liquid sampler (Bioaerosol fast sampler: BIAFTS) able to collect the finest airborne particles into a liquid sample, typically in 2–3 min, at a flow rate flow of 1.44 m^3^/min was calibrated and validated with a bacteriophage ϕ29 model by comparison with an already validated filtration-based method with polytetrafluorethylene (PTFE) filters (30 min–2 h, 0.3 m^3^/min) with a described efficiency between 93% and close to 100% [19,20].

While aerobiological studies using hazardous viruses can provide valuable information, they can also expose personnel to unnecessary risks. Surrogate viruses, such as the 35 nm-sized ϕ29 bacteriophage, one of the smallest and simplest known dsDNA phages, that can be used to mimic the behaviour of the finest pathogenic viruses [15,21]. 

The aim of this study goes further in testing the capability of Counterfog^®^ BIAFTS of capturing naturally emitted airborne pathogens, in particular, real SARS-CoV-2 aerosols generated by COVID-19 patients’ in hospital rooms.

## 2. Materials and Methods

### 2.1. Sample Collection 

Aerosol samples were collected using two different air sampling technologies. The first technology is a filtration system through polytetrafluorethylene (PTFE) filters connected to a vacuum pump. PTFE filters can collect particles at high efficiency, maintaining the recovery efficiency of viable virus [19]. 

**Figure 1:**
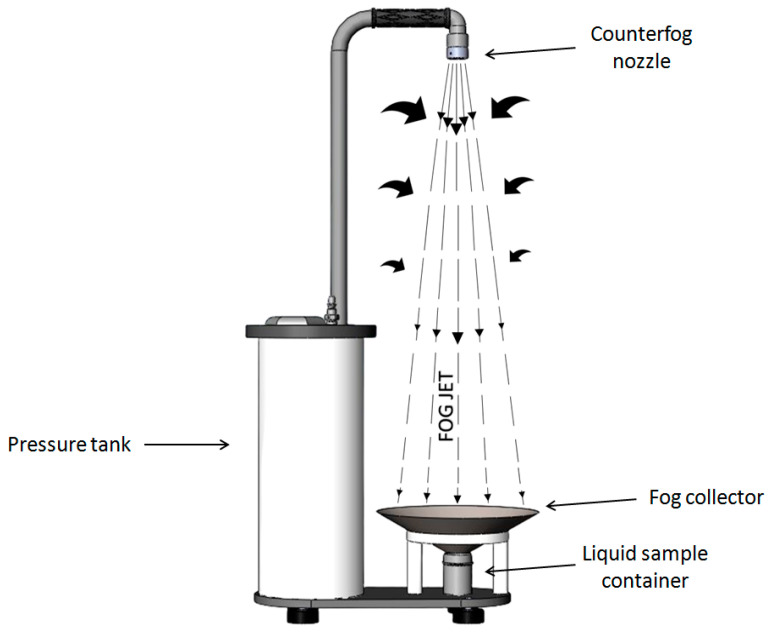
Operating principle of the Counterfog BIAFTS technology.

The second technology is the Bioaerosol Fast Sampler (BIAFTS) (COUNTERFOG S.L., Madrid, Spain), a device that collects airborne particles into a liquid medium. It is based on Counterfog technology: dynamic fog cones of liquid nanometric droplets that when projected, capture agents transported in the air [22]. BIAFTS has been developed under H2020 INNO4COV-19 project for the fight against COVID-19 pandemic (Figure 1).

### 2.2. Aerosolization and Sampling of Test Bacteriophage ϕ29 

Prior to the field sampling for the SARS-CoV-2 aerosol samples, the integrity and robustness of the experimental protocol was examined in the laboratory and in a relevant environment. Virus aerosol collection by the PTFE filters and BIAFTS technologies was first tested using the ϕ29 virus as a surrogate.

In our laboratory tests, 2.5 × 10^11^ pfu of bacteriophage ϕ29 were diluted in 200 mL of phage diluent solution (50 mM TrisClH pH 7.8, 10 mM MgCl_2_, 100 mM NaCl, 0.05% Tween-20) and placed in the container to be aerosolized with the Counterfog nozzle and dispersed in a 25 m^3^ room. The nebulization and dispersion process was performed three times, and a sampling method was employed for the recovery of bacteriophage ϕ29 aerosols. In the first one, PTFE filtration-based method (30 min, 0.3 m^3^/min), already validated for viral capture in air, was used for sampling. In the other two, the BIAFTS system was calibrated for optimal recovery of airborne viruses in liquid samples. Two different measures were taken after each ϕ29 nebulization; different system features of pressure, liquid flow and aspirated air volume, as well as sampling time were tested to establish the optimal capture characteristics. Both samplers were placed approximately at a 1 m height. PTFE filters were collected in tubes containing 2 mL of phage diluent solution; and BIAFTS air samples were collected in 50 mL Falcon tubes using phage diluent solution. Samples were kept at 4 °C until sample processing.

**Figure 2:**
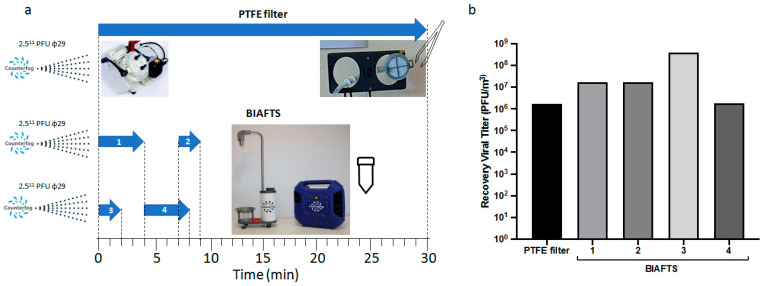
Bacteriophage ϕ29 aerosols recovery in calibration tests in a 25 m^3^ room. Artificially generated bacteriophage ϕ29 aerosols are captured using PTFE filtration-based sampler and BIAFTS. (**a**) Sampling timeline after the three ϕ29 dispersion experiments. (**b**) Viral titer recovered in each of the air samples (PFU/m^3^).

Relevant environment tests were performed in a standard railway car. An aliquot of 7.6 × 10^11^ pfu of bacteriophage ϕ29 was diluted in 1 L of phage diluent solution (50 mM TrisClH pH 7.8, 10 mM MgCl_2_, 100 mM NaCl, 0.05% Tween-20) and placed in the container to be nebulized by the Counterfog nozzle and dispersed through the whole railway car (156.47 m^3^). Three air pumps coupled to PTFE filters were distributed along the railway car: two at the ends and one in the center. Liquid samples with BIAFTS were taken at one side of the railway car (Figure 2a). Both samplers were placed at approximately a 1.5 m height. After nebulization, PTFE filtration systems were started and in-liquid samples were simultaneously taken with the BIAFTS system. Air samples were taken at different times for 1 h after nebulization: PTFE filters (10 min, 0.3 m^3^/min) at 0, 15, 30, and 50 min; and BIAFTS (2 min, 1.4 m^3^/min) at 5, 20, 35, and 55 min. PTFE filters were collected in tubes containing 2 mL of phage diluent solution; and air samples from BIAFTS were collected in 50 mL Falcon tubes using phage diluent solution. Samples were kept at 4 °C until sample processing. 

Additionally, the number and size of the particles was monitored every second by a particle counter with a 10% mass concentration precision (Sensirion Particle sensor SPS30, Sensirion, Stäfa, Switzerland). This device was place next to the central door of the railway car at an approximate height of 0.6 m.

### 2.3. Field Tests with SARS-CoV-2

After testing the technologies with artificial aerosols of ϕ29 surrogate, real field tests were conducted. SARS-CoV-2 samples were collected, using both technologies, in rooms of COVID-19 patients from Hospital Universitario Fundación Alcorcón in Madrid during the Omicron variant wave. The hospital rooms were approximately 80 m^3^, including the bathroom. Each PTFE filters’ sampling lasted for 1 h 45 min to 2 h, and the filters were collected in tubes containing 2 mL of phosphate-buffered saline (PBS) containing 10 µg/mL bovine serum albumin (BSA); 100 µg/mL gentamicin; 200 U/mL penicillin, and 20 µg/mL streptomycin. Air samples from BIAFTS were collected in 2–4 min in 50 mL Falcon tubes using PBS medium supplemented with 0.1% BSA, 100 µg/mL gentamicin, 200 U/mL penicillin, and 20 µg/mL streptomycin. Samples were kept at 4 °C until sample processing.

Air samples were collected in six hospital rooms and two hospital bathrooms from patients of the COVID area of the hospital. Each room was occupied by two patients of recent infection (less than 3 days post-infection). Both systems were placed in the middle of the room at a considerable distance from patients’ beds. 

### 2.4. Analytical Methods

Samples of ϕ29 were taken to the laboratory to be analyzed. To determine the amount of virus particles in the samples, the plaque assay titration method on *Bacillus subtilis* was employed. When a suspension of an infective phage, in this case ϕ29, is spread over the lawn of susceptible bacterial cells, the lysis plaques can be seen over the bacterial lawn. For each sample, serial dilutions (1/10) were made. Each of them was titered in duplicate following the two-step double-agar overlay method [23]. In these assays, the plating was done in 6-well plates. A negative control with no virus was sampled in one of the wells. The plates were incubated at 37 °C (approximately 24 h). After incubation, plaque-forming units (PFU) were manually counted.

After the collection of SARS-CoV-2 aerosol samples in hospital rooms, all samples were processed immediately in the BSL-3 laboratory of CBMSO (CSIC-UAM). The different volumes of supplemented PBS containing the air samples from BIAFTS were first concentrated using Amicon^®^ Ultra-15 50 K up to approximately 2 mL, and 350 µL were mixed with one volume of Lysis buffer (Promega, Madison, WI, USA). On the other hand, the PTFE samples were vigorously vortexed and 350 µL were also diluted 1:1 in Lysis buffer as mentioned before. Viral RNA isolation from all samples was performed from 600 µL, after adding 30 µL of proteinase K, following Maxwell^®^ RSC Viral TNA kit instructions, in a Maxwell^®^ RSC 48 Instrument (Promega) isolation. RNA was eluted in 50 µL of nuclease free water. In every RNA extraction, a blank was included to check possible contamination during sample manipulation.

Finally, the presence of SARS-CoV-2 was determined by RT-qPCR in a CFX Opus 384 Real Time PCR system (BIORAD). Reactions were performed in 20 µL, with 4 µL of undiluted RNA, including 1x of Reliance One-Step Multiplex Master mix (BIORAD) and 500–900 nM and 250 nM of N-gene specific primers and probes (CDC sequences: N2 forward 5′-TTACAAACATTGGCCGCAAA-3′, N2 reverse 5′-GCGCGACATTCCGAA GAA-3′ and N2 probe 5′-FAM-ACAATTTGCCCCCAGCGCTTCAG-3′; N1 forward 5′-GACCCCAAAATCAGCGAAAT-3′, N1 reverse 5′-TCTGGTTACTGCCAGTTGAAT CTG-3′ and N1 probe accccgcattacgtttggtggacc-3′). The amplification protocol included 10 min at 50 °C followed by 10 min at 95 °C and 40 cycles of 10 s at 95 °C and 30 s at 60 °C. A standard curve was built with SARS-CoV-2 synthetic RNA (Twist BioScience), and a non template control was included in each plate. Measures were performed in triplicate.

### 2.5. Data Analysis

The data were extracted and analyzed in a spreadsheet developed in Excel software, version 2007 (Microsoft, Redmond, WA, USA). Real Time PCR data was processed and analyzed using CFX Maestro 2.2 (BioRAD, Hercules, CA, USA)).

### 2.6. Calculation of Bacteriophage ϕ29 Aerosol Concentration

The aerosol concentration in calibration tests with bacteriophage ϕ29 was calculated as follows:

(1) $$\mathrm{Filter}\;\mathrm{and}\;\mathrm{BIAFTS}\;\mathrm{samples}\;(\mathrm{PFU}/m^{3})=\frac{\left[C\right]\;\left(PFU/mL\right)\;\times \;\begin{array}{c} Total\\ \;volume\;\left(mL\right) \end{array}}{Time\;\left(min\right)\;\times \;Sampling\;rate\;\left(m^{3}/min\right)}$$

Total volume refers to the total expected liquid volume recovered using the manufacturer specifications for liquid flow (Counterfog^®^) in the case of BIAFTS samples; and 2 mL for filter samples. Aspirated air volume or sampling rate is also indicated in the specifications: 1.4 m^3^/min for BIAFTS and 0.3 m^3^/min for the filtering system.

## 3. Results

### 3.1. Calibration Tests of BIAFTS Using Bacteriophage ϕ29

Calibration of BIAFTS and its efficiency was tested using artificially generated aerosols of bacteriophage ϕ29 in a 25 m^3^ room (experimental design is explained in more detail in the Materials and Methods). The initial amount of ϕ29 to be dispersed was 2.5 × 10^11^ plaque forming units (PFU). The generated fog, created with Counterfog^®^ technology, was calculated to contain an average of 1 × 10^7^ ϕ29 PFU/m^3^. Three ϕ29 nebulization and dispersion processes were performed, and air samples were taken with PTFE filters and BIAFTS (Figure 2a). Both methodologies were comparable and capable of capturing around 6 and 7 logs PFU/m^3^ of bacteriophage ϕ29, which is translated into a high capture and sampling efficiency (Figure 2b). Sample BIAFTS 3 recovered 8 logs of PFU/m^3^, this might be due to a less homogeneous dispersion of the virus in this run.

### 3.2. Bacteriophage ϕ29 Aerosol Evolution over Time

**Figure 3:**
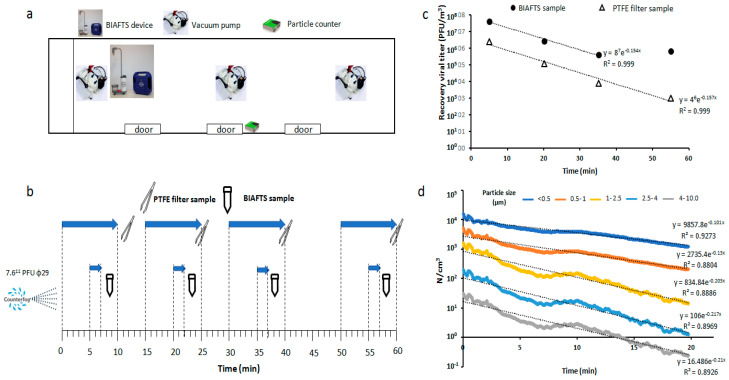
Bioaerosol dynamics and capture tests in a railway car. (**a**) Railway car diagram that shows the space distribution of air samplers and particle counter. (**b**) Timeline and experimental design of bioaerosol tests. A unique bacteriophage ϕ29 aerosolization was performed in a 157 m^3^ railway car, and samples were taken over the course of 1 h. Sampling time: 2 min for BIAFTS and 10 min for PTFE filters. (**c**) Viral titer of bacteriophage ϕ29 (PFU/m^3^) recovered using BIAFTS and PTFE filtration. (**d**) Natural aerosol decay measured in number of particles/cm^3^ for different particle sizes: ≤0.5 µm (dark blue), 0.5–1 µm (red), 1–2.5 µm (yellow), 2.5–4 µm (light blue), and 4–10 µm (grey).

Aerosol dynamics depends on the particle size, with smaller particles remaining longer in air. In line with previous assays, tests with ϕ29 aerosols were performed in a 157 m^3^ railway car with no ventilation system to compare biological aerosols’ and different particle sizes’ decay to establish the main droplet size of bacteriophage ϕ29 aerosols (Figure 3). Several bioaerosols samples were taken using both the BIAFTS and PTFE filtering system over the course of 1 h (Figure 3b). Both systems showed similar efficiencies for capturing ϕ29 bioaerosols, and the same aerosol decay was observed (Figure 3c) (Table S1).

The number and size of the particles was monitored by a particle counter (Sensirion Particle sensor SPS30). Figure 3d shows the decay as a function of each particle size. The decay is proportional to the particle size. Then, the bigger the particles, the higher the slope of their logarithmic decay in time. Comparing the results in Figure 3d with the ϕ29 aerosols’ slope in Figure 3c, it appears that the generated ϕ29 aerosols decayed according to a droplet size range of 0.5–1 µm. The decay value suggests that the BIAFTS is capable of capturing particles of at least 0.5–1 µm. Other research papers show that this technology is capable of capturing particles of diesel dust of 0.3 µm which is a great advance due to the small sizes of viruses [24].

Some environmental sampling and laboratory experiments examined the particle size distribution of SARS-CoV-2 in the air, obtaining diverse results probably due to different environmental conditions. Respiratory viruses in the air are likely to be found in water droplets much larger than the viruses themselves [25]. A study performed in hospital rooms reported SARS-CoV-2 PCR-positive particles of sizes >4 μm and 1–4 μm, but negative for samples from the fractionated size <1 μm [16]. However, another study from Wuhan, China detected SARS-CoV-2 in aerosols 0.25–1.0 μm in diameter [17].

### 3.3. Real SARS-CoV-2 Bioaerosols Detection in Hospital Rooms

**Figure 4:**
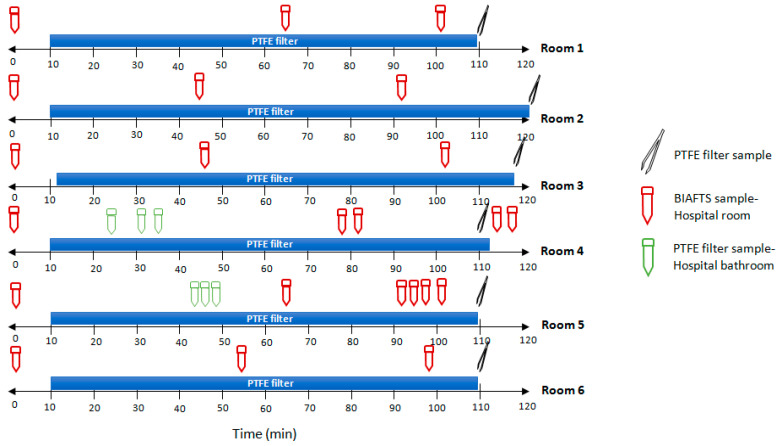
Samples collection timeline of hospital rooms tests. Air samples from COVID-19 hospital rooms were taken in a 2-h time interval. SARS-CoV-2-containing aerosols from six different rooms were sampled using PTFE filters and BIAFTS. PTFE filters samples were taken for 105–120 min, and BIAFTS samples were taken in 2 min at different times in the same time interval. Blue bars indicate the duration of PTFE filtration process; red falcon symbols-BIAFTS samples taken in the hospital room; and green falcon symbols-BIAFTS samples taken in the bathroom of the corresponding hospital room. Each hospital room was occupied by two COVID-19 patients.

In order to transfer this technology to real world settings and to sample natural biological aerosols, tests were performed in hospital rooms (Hospital Universitario Fundación Alcorcón) with COVID-19 patients. SARS-CoV-2 viral loads peak in the first week of infection, therefore the presence of SARS-CoV-2 in the air is possibly highest in the first week of illness [16]. For this reason, tests were performed in hospital rooms with recently infected patients. The results show the detection of SARS-CoV-2 in the air using both systems simultaneously (Table 1). Air samplers were located in the middle of the room, at a distance of at least 2–3 m from the patients. The concentration of SARS-CoV-2 in air is low, with high Ct values after RT-qPCR analysis/detection. PTFE filters allowed us to see the average of virus concentration in a period of 2 h, whereas BIAFTS is a fast sampler that allows punctual sampling in only a few minutes (Figure 4). With both systems, we obtained comparable results (Table 1). Results from each room are different because patients and the aerosol emissions were different. We can observe rooms with no or very low SARS-CoV-2-containing aerosols concentration (rooms 4 and 6) and others with higher levels of SARS-CoV-2 in aerosols (room 5). Interestingly, levels of SARS-CoV-2-containing aerosols are similar in bathrooms, where at the moment of sampling, there were no patients inside, and rooms (rooms 4 and 5), showing the aerosol transport through the air. Surprisingly, results obtained from BIAFTS samples show that there are sudden boosts in SARS-CoV-2-containing aerosols, detecting moments of high concentration of viral genome. This is especially striking in rooms 1 and 2 (Table 1). Ventilation and air circulation in the hospital room has to be taken into account. In the case of rooms in Hospital Universitario Fundación Alcorcón, there are two air replacements/hour. This corresponds with a virus reduction, if there were no other emission, of 0.868 log/hour, calculated using the following equation:

(2) $$\frac{d\left[C\right]}{dt}=\frac{-q\;\times \;\left[C\right]}{V}$$

where [*C*] is the SARS-CoV-2 concentration in air; *q* is the airflow of ventilation; and *V* is the volume of the hospital room. 

The air extraction of the ventilation system is located in the bathroom, where the air is directed for recirculation and renewal, which also explains the levels of virus concentration in the bathrooms. 

## 4. Discussion

In this study, we tested hospital rooms occupied with COVID-19 patients using a new air sampling device, BIAFTS, for the rapid capture of airborne pathogens in a liquid medium. The results obtained with BIAFTS are very promising since similar results to those generated with other air-sampling technologies, such as PTFE filtration, were obtained in much less time. The technology we have developed has several advantages: it allows surveillance of a large volume of air in a short period of time and offers the possibility of understanding the dynamics of aerosols as it can detect aerosol levels at very specific moments.

Based on the results obtained in hospital rooms, it seems that SARS-CoV-2 concentration levels in aerosols are sudden and highly localized in the room. Thanks to the calibration performed with bacteriophage ϕ29 and to the comparison with the PTFE-based filtration system (validated for virus capture in air) [19], we know that BIAFTS captures, with a high efficiency, the virus-containing aerosols that are present at that specific moment. We can reach the conclusion that the distribution of SARS-CoV-2-containing aerosols is not homogeneous in the hospital room. This is also shown in other airborne particles studies [26]. Bursts of highly virus-loaded aerosols occurred at specific moments, and this could be due to ventilation processes or to the release of infective aerosols by patients that might occur in bursts of varying concentrations and sizes. By contrast, aerosol distribution in the railway coach tests was very homogeneous as these tests were performed without the ventilation system working and a unique homogeneous artificial virus release event. SARS-CoV-2 airborne distribution is more homogeneous in the bathroom; this makes sense as the airflow is directed there, mixing and diluting any agent present in aerosols. Another aspect to take into account, regarding the virus levels detected in aerosols, is the cleaning capacity of BIAFTS as it captures and collapses airborne agents, removing them from the air with an airflow of 1.44 m^3^/min.

There are some studies that have detected SARS-CoV-2 RNA in airborne material collected by air samplers [16,17,18]. From all the existing air sampling technologies, filters are generally the most efficient for determining viral loads in aerosols, but they cause more damage to viruses during the process than the other methods [15]. Any respiratory virus that can survive aerosolization implies an inhalation biohazard risk; however, not all the studies detecting SARS-CoV-2 genetic material have demonstrated its infectivity; in part because of the difficulties in sampling virus-containing aerosols in real settings and the presence of low viral concentrations, impeding the growth of viruses in cell cultures. Only Lednicky et al (2020) had been able to sample viable SARS-CoV-2 from air in the hospital environment [2] demonstrating that this virus remains infectious in the air. Another study established between 3–16 h of infectivity of SARS-CoV-2 under experimental conditions [27,28]. The methodology employed for sampling in Lednicky et al. work is the VIVAS air sampler (three-hour sampling), that collects virus particles without damaging them, thus conserving their viability. This sampler operates using a water vapor condensation mechanism. The collection of bioaerosols into a liquid medium helps the conservation of viability because it prevents the virus from drying out and becoming inactivated. BIAFTS has the advantage of capturing bioaerosols in any liquid medium in a very short time. However, we had some difficulties in growing the virus in cell culture due to the low virus concentration and the presence of other contaminants in the recovered sample that seems to be toxic for these cells.

Our study has some limitations that should be taken into account: the main problem of environmental air samples is the low-virus concentration present in the air that, in some cases, can be close to the limit of detection of the detection technique. Some obtained results are indeterminate, and new samples are recommended, which in this case has not been possible. The low concentration, the air sampling stressors, and the rest of the contaminants recovered in the air sample are some of the causes that make the culture of viable virus very difficult but needed for determining the infectiousness of the detected particles. Finally, the clinical characteristics of COVID-19 patients have not been considered, and although all patients were in the first 3 days of infection, not all of them will produce the same amounts of aerosols.

## 5. Conclusions

In conclusion, the air-to-liquid sampler presented in this work is an effective and fast tool for capturing airborne matter, including bioaerosols, in a very short time, which allows fast decision-making when detecting any kind of hazard in the air. It is also useful for a better understanding of aerosols dynamics and bioaerosol formation thanks to its capacity of sampling large air flows.

It is a revolutionary sampler due to the large volume of air it collects and to the aerosol-capture capability compared to other methodologies, such as filtering systems. Furthermore, sampling bioaerosols in a liquid medium can be very helpful for maintaining viability and studying viral infectivity.

Finally, the results of sampling aerosols in rooms with COVID-19 patients, all wearing masks, show the presence of SARS-CoV-2 aerosols. Additionally, sudden boosts in SARS-CoV-2 aerosols emissions were found, suggesting that SARS-CoV-2 could be released abundantly at certain moments. 

## 6. Patents

José Luis Pérez-Díaz and Antonio Alcamí are co-inventors of a patent application on BIAFTS.

## Figures and Tables

**Table 1:** RT-qPCR detection of SARS-CoV-2 RNA in aerosols from hospital rooms. The results are presented in RNA copies/m^3^ air (N-gene), detection obtained by RT-qPCR, mean Ct from triplicates. Standard curve: y = -3.348 + 38.2; R^2^ = 0.987; Efficiency = 98.9%; curve between 2 × 10^3^ and 1 copies of synthetic virus RNA per well. Result interpretation as “indeterminate” refers to those samples with Ct > 40 for one of the N-genes (N1 or N2). All BIAFTS samples were taken in 2 min and PTFE filter samples in 1 h 45 min−2 h (Figure 3a).

Room	Ct Value	Result	Airborne SARS-CoV-2 (RNA Copies m^−3^ air)	Sampler Used
1	-	Negative	ND	BIAFTS
	33.49	Positive	547.09	
	-	Negative	ND	
	34.10	Positive	258.45	PTFE filter
2	31.04	Positive	4235.00	BIAFTS
	39.02	Indeterminate	9.55	
	-	Negative	ND	
	35.06	Positive	234.45	PTFE filter
3	36.53	Positive	82.66	BIAFTS
	36.10	Positive	146·28	
	36.54	Positive	99.00	
	33.64	Positive	650.74	PTFE filter
4	36.45	Indeterminate	5.10	BIAFTS
	35.83	Positive	14.39	
	36.31	Positive	6.93	
	35.71	Positive	2.31	
	-	Negative	ND	
	34.47	Positive	148.15	PTFE filter
Bathroom-Room 4	36.6	Positive	3.91	BIAFTS
	35.9	Positive	10.89	
	37.7	Indeterminate	3.63	
5	36.45	Positive	197.42	BIAFTS
	37.36	Positive	172.40	
	37.56	Indeterminate	132.08	
	37.64	Indeterminate	183.73	
	36.85	Positive	250.38	
	34.17	Positive	157.68	PTFE filter
Bathroom-Room 5	38.32	Positive	272.06	BIAFTS
	38.22	Indeterminate	230.39	
	38.96	Indeterminate	125.00	
6	-	Negative	ND	BIAFTS
	-	Negative	ND	
	-	Negative	ND	
	34.33	Positive	456.96	PTFE filter

## Data Availability

Not applicable.

## Supplementary Materials

The following supporting information can be downloaded at: https://www.mdpi.com/article/10.3390/ijerph20010576/s1, Table S1. This table shows the presence of infectious ϕ29 (pfu) in air in a railway train at different times after artificially generating the aerosol.
