# Supplementary material for: Fast Air-to-Liquid Sampler Detects Surges in SARS-CoV-2 Aerosol Levels in Hospital Rooms
Source: Int J Environ Res Public Health. 2022 Dec 29;20(1):576. doi: 10.3390/ijerph20010576 (PMC9819203; doi:10.3390/ijerph20010576)
Supplement: Supplementary file 1 [file ijerph-20-00576-s001.zip › ijerph-2087531-supplementary.pdf]

Supplementary Materials

Table S1.
Presence of infectious ϕ29 (pfu) in air in a railway train (156.47 m^3) at different times after artificially generating the aerosol. Air samples collected by PTFE filters and fast air sampler **BIAFTS**.

	**CONTROL**				**T 0-10 min**			
	**1**	**2**	**3**	**BIAFTS control**	**1**	**2**	**3**	**BIAFTS 5 min**
Viral titer (pfu/ml)	0	0	0	0	$4{\cdot}25 \times 10^5$	$3{\cdot}80 \times 10^5$	$3{\cdot}25 \times 10^5$	$4{\cdot}15 \times 10^5$
Viral titer per volume (pfu)	0	0	0	0	$8{\cdot}50 \times 10^5$	$7{\cdot}60 \times 10^5$	$6{\cdot}50 \times 10^5$	$1{\cdot}25 \times 10^8$
Viral titer/ m^3	0	0	0	0	$2{\cdot}83 \times 10^6$	$2{\cdot}53 \times 10^6$	$2{\cdot}17 \times 10^6$	$4{\cdot}15 \times 10^7$
Virus in the railway car (pfu)	0	0	0	0	$4{\cdot}43 \times 10^8$	$3{\cdot}96 \times 10^8$	$3{\cdot}39 \times 10^8$	$6{\cdot}49 \times 10^9$

	**T 15-25 min**				**T 30-40 min**			
	**1**	**2**	**3**	**BIAFTS 20 min**	**1**	**2**	**3**	**BIAFTS 35 min**
Viral titer (pfu/ml)	$1{\cdot}75 \times 10^4$	$1{\cdot}90 \times 10^4$	$1{\cdot}60 \times 10^4$	$2{\cdot}75 \times 10^4$	$6{\cdot}40 \times 10^2$	$1{\cdot}65 \times 10^3$	$1{\cdot}25 \times 10^3$	$4{\cdot}10 \times 10^3$
Viral titer per volume (pfu)	$3{\cdot}50 \times 10^4$	$3{\cdot}80 \times 10^4$	$3{\cdot}20 \times 10^4$	$8{\cdot}25 \times 10^6$	$1{\cdot}28 \times 10^3$	$3{\cdot}30 \times 10^3$	$2{\cdot}50 \times 10^3$	$1{\cdot}23 \times 10^6$
Viral titer/ m^3	$1{\cdot}17 \times 10^5$	$1{\cdot}27 \times 10^5$	$1{\cdot}07 \times 10^5$	$2{\cdot}75 \times 10^6$	$4{\cdot}27 \times 10^3$	$1{\cdot}10 \times 10^4$	$8{\cdot}33 \times 10^3$	$4{\cdot}10 \times 10^5$
Virus in the railway car (pfu)	$1{\cdot}83 \times 10^7$	$1{\cdot}98 \times 10^7$	$1{\cdot}67 \times 10^7$	$4{\cdot}30 \times 10^8$	$6{\cdot}68 \times 10^5$	$1{\cdot}72 \times 10^6$	$1{\cdot}30 \times 10^6$	$6{\cdot}42 \times 10^7$

	**T 50-60 min**			
	**1**	**2**	**3**	**BIAFTS 55 min**
Viral titer (pfu/ml)	$1{\cdot}55 \times 10^2$	$1{\cdot}98 \times 10^2$	$1{\cdot}05 \times 10^2$	$6{\cdot}65 \times 10^3$
Viral titer per volume (pfu)	$3{\cdot}10 \times 10^2$	$3{\cdot}95 \times 10^2$	$2{\cdot}10 \times 10^2$	$2{\cdot}00 \times 10^6$
Viral titer/ m^3	$1{\cdot}03 \times 10^3$	$1{\cdot}32 \times 10^3$	$7{\cdot}00 \times 10^2$	$6{\cdot}65 \times 10^5$
Virus in the railway car (pfu)	$1{\cdot}62 \times 10^5$	$2{\cdot}06 \times 10^5$	$1{\cdot}10 \times 10^5$	$1{\cdot}04 \times 10^8$
